# Investigating the sequential patterns of methamphetamine use initiation in Iran

**DOI:** 10.1186/s13011-020-00297-z

**Published:** 2020-07-29

**Authors:** Ebrahim Moghimi Sarani, Jamshid Ahmadi, Bahare Oji, Motahareh Mahi-Birjand, Nader Bagheri, Amir Bazrafshan, Marie Dehghan Manshadi, Sajad Yaghoubi, Asiyeh Dezhkam, Mehrdad Khatami, Meghdad Abdollahpour-Alitappeh

**Affiliations:** 1 Research Center for Psychiatry and Behavior Science, Shiraz university of Medical Sciences, Shiraz, Iran; 2 Substance Abuse Research Center, Dual Diagnosis Ward, Shiraz University of Medical Sciences, Shiraz, Iran; 3 Department of Psychiatry, Psychosomatic Medicine Research Center, Tehran University of Medical Sciences, Tehran, Iran; 4 Infectious Disease Research Center, Birjand University of Medical Sciences, Birjand, Iran; 5 Department of Clinical Pharmacy, Faculty of Pharmacy, Birjand University of Medical Sciences, Birjand, Iran; 6 Cellular and Molecular Research Center, Basic Health Sciences Institute, Shahrekord University of Medical Sciences, Shahrekord, Iran; 7 Department of Humanistic Sciences, Islamic Azad University of Yazd, Yazd, Iran; 8 Department of Clinical Microbiology, Iranshahr University of Medical Sciences, Iranshahr, Iran; 9 Department of Midwifery, School of Nursing and Midwifery, Iranshahr University of Medical Sciences, Iranshahr, Iran; 10 NanoBioelectrochemistry Research Center, Bam University of Medical Sciences, Bam, Iran; 11 Cellular and Molecular Biology Research Center, Larestan University of Medical Sciences, Larestan, 7431989335 Iran

**Keywords:** Methamphetamine (MA), Methamphetamine use patterns, Methamphetamine use initiation, Substance sequences

## Abstract

**Background:**

Methamphetamine (MA) remains one of the most commonly used amphetamine-type stimulants, accounting for the second most widely-used substance after marijuana. Due to increased use of MA, a wide variety of research has focused on the patterns of MA use initiation among adolescents. Nevertheless, there are few data available for people who use MA. The present study set out to assess the sequential patterns of substance use initiation in patients with MA use disorders in Iran.

**Materials and methods:**

This cross-sectional study described substance initiation patterns for 302 patients who used MA admitted to hospitals and psychiatric centers of Shiraz University of Medical Sciences. The study was conducted between April 2015 and June 2016. After obtaining informed consents, participants were interviewed by trained interviewers using face-to-face, semi-structured interviews. The collecting data were analyzed using the chi square tests and one-way analysis of variance (ANOVA) tests to compare the relationship between qualitative and quantitative variables, respectively.

**Results:**

Out of 302 participants enrolled in the study, 16 (5.3%) and 286 (94.7%) were female and male, respectively. The mean age of participants in the study was 37.29 years. The mean age of onset of MA use was found to be 15.9 years. 46.1% of the patients started MA use before 15 years. 77.2% of the patients who used MA had family members with a history of substance use. 93.71% of the patients who used MA started substance use with tobacco, alcohol, or opium, as the most frequent substances. Tobacco, as the first substance or starting substance, exhibited the most widely-used substance (69.53% of the cases). Tobacco-alcohol-cannabis-opium-heroin-MA sequencing was significantly related to the early onset of the substance use. Early-onset substance use was significantly higher in those with lower income, primary education, and family history of substance use. No significant relationship was found between employment status with the age of onset of substance use, and different substance use with marital status.

**Conclusion:**

Tobacco, alcohol and opium can be considered as the main sequencing substances for initiation to MA use. Standardized measures to decrease and control access to main starting and sequencing substances, including tobacco, alcohol, and opium, can greatly help decrease the early onset of the MA use, develop suitable prevention, and establish early intervention strategies.

## Introduction

Amphetamines, as sympathomimetics and psychostimulants, are the most commonly-used drugs after cannabis in the UK, Australia, and several Western European countries. Methamphetamine (MA), a potent form of amphetamine first made in 1893 by Japanese researcher Nagai Nagayoshi 6 years after the discovery of amphetamine, has been one of the most commonly used amphetamine-like drugs in recent years [1]. MA is a highly addictive and euphoric stimulant, representing one of the largest illegal drugs in the world which has become more prevalent than other amphetamine derivatives [2, 3]. Generally, the use of amphetamine-like stimulants, including MA, is a major concern, representing the second most commonly used substance after marijuana according to the United Nations Office on Drugs and Crime [2]. In the United States, the number of patients who use MA increased from 353,000 in 2010 to 569,000 in 2014 [4].

MA is used through a variety of routes, including inhalation, smoking, intravenous injection, and oral intake [4]. This white crystalline drug is absorbed quickly from the mouth and its effects usually appear within an hour after ingestion [2]. MA releases and inhibits the reabsorption of serotonin, norepinephrine, and dopamine. The high levels of such neurotransmitters in synapses lead to euphoria, insomnia, increased libido as well as increased energy for a longer time. Short-term adverse effects include increased blood pressure, heart rate and respiratory rate, chest pain, decreased appetite, anorexia, restlessness, confusion, tremor, seizure, anxiety, aggression, and symptoms of psychosis, including delusions and paranoia. Because of its ready availability, inexpensiveness and reinforcing properties, the chronic use of MA can have serious and potentially dangerous consequences [5].

MA is a heavily-abused substance with an estimated 24 million users worldwide, indicating a serious and growing public health issue in a variety of countries [6]. Although decreasing significantly from ∼ 35 million in 2005, the prevalence of MA use has increased in Europe and Asia, in countries such as the Netherlands, China, India, and Iran [6–8]. Of note, Iran faces with a higher rate of substance use as compared with many countries in the world, so that the rate of substance use has tripled in Iran in recent years as compared with the rate of population growth [9–13]. Although opium and cannabis are considered as traditional drugs of abuse in Iran, the use of heroin, crystal, ecstasy, and MA has increased in recent years [14]. In 2010, the Persian police reported a 50% decrease and 54% increase in seizing opiates and MA, respectively, as compared with 2009 [15]. It is estimated that there are 1.2–2 million users who are dependent on illicit drugs mainly opiates and MA. Epidemiological studies revealed the prevalence of MA use in 7, 73.9 and 13.3% of the adults in the general population of Tehran [16], truck drivers across the country [17] and male bodybuilders in Tehran [18], respectively. Increased physical energy, wakefulness, pleasure-seeking and professional performance were found to be the main reasons for MA use [15]. Analyses of substance use initiation and sequencing patterns may help find risk factors for other behaviors.

Considering the increased prevalence of the stimulant use such as MA in Iran, there is an urgent need to study the sequence and profile of substance use before MA use in Iran. Therefore, the present study was tailored to evaluate the sequential patterns of substance use initiation in patients with MA use disorders in Iran.

## Materials and methods

### Ethics statement

This study was carried out in accordance with the recommendations of ethical guidelines, IR.SUMS.REC.1394.S1206. The research protocol was approved by Shiraz University of Medical Sciences. All subjects were given written informed consent in accordance with the Declaration of Helsinki.

### Design

This cross-sectional study was conducted between April 2015 and June 2016 to investigate the substance use in the patients who used MA and to find the sequential patterns of MA use. Our sample consisted of all patients who used MA admitted to hospitals and psychiatric hospitals affiliated to Shiraz University of Medical Sciences, including Ibn-e-Sina Psychiatric Educational Hospital, Hafez Educational Hospital, and Recovery Center during the study period.

### Participants

In the centers that the samples were collected, all the people used substances and almost all the patients used MA. Interviews were conducted by a psychologist or assistant psychologist. The participants were evaluated at one-time point by face-to-face, semi-structured interviews for approximately 20–30 min. Those patients who met DSM5 diagnostic criteria for MA use disorders were included in the study. Only a small number of the patients who did not meet these criteria were excluded from the study (such as those who did not sign the consent form or lacked a good memory). In total, 302 patients (95% confidence coefficient and 5% error and alpha = 0.05) were participated in this study. The study followed the Strengthening the Reporting of Observational Studies in Epidemiology (STROBE) guidelines [19] to strengthen the methodology and report of the findings.

### Inclusion criteria

Potential participants were eligible for the study if they met the following inclusion criteria:

- Age between 18 and 65 years old
- MA use over the past year based on DSM5 criteria
- DSM5 criteria for MA use disorders

### Exclusion criteria

Patients were excluded from the study if they met one of the following exclusion criteria:

- People with cognitive impairment, severe aggression, and psychosis who were unable to cooperate in the interview and whose information was not sufficiently reliable at the time of the interview
- Discontent to participate in the study

### Procedure

After obtaining written informed consent, qualified patients were interviewed and the required information was collected on the basis of a pre-prepared checklist. In-person interview checklist included:

- Demographic information including age, sex, marital status, occupation, income, and education
- The history of different types of substance use in the past year
- The age of the first substance use
- The first substance used
- Sequential use of substances from beginning up to now (to find sequential patterns)
- The history of substance uses in the family, the number of family members with substance use, and the type of substances used
- Previous history of admission to Psychiatric Hospital

Substances used were classified according to substance use and drug classification as follows:

- The tobacco group: including hookah and cigarettes
- The alcohol group: only including alcohol
- The cannabis group: including marijuana and cannabis
- The opium group: including opium and opium sap
- The heroin group: including heroin and crack
- The cocaine group: only including cocaine
- The Ritalin group: only including Ritalin
- The MA group: only including MA

### Sequential profiling

The sequential patterns for MA use were determined based on the order of substance use in patients who used MA. In the questionnaire, we asked participants specifically about the order of the substances that they used before MA use.

### Statistical analysis

The data were evaluated by SPSS software version 20. The results were analyzed by descriptive statistics, including mean ± standard deviation for quantitative variables and frequency tables for qualitative variables. In addition, chi-square and one-way analysis of variance (ANOVA) tests were used to compare the relationship between qualitative and quantitative variables, respectively. If the result of the ANOVA test was significant, a least significant difference test was applied to locate which of the means differed. *P* values < 0.05 were considered to be statistically significant.

## Results

A total of 302 participants were enrolled in the study. Table 1 indicates demographic characteristics of the sample, including gender, age, education, monthly income, age of onset of substance use, history of hospitalization, the rate of substance use in the family, type of substances used in the family member, and type of substances used in the last year. The mean age of participants in the study was 37.29 years and the mean age of onset of substance use was found to be 15.9 years.

**Table 1:** Demographic characteristics of the study population

Characteristics		Number (*n* = 302)	Percent
Gender	Male	286	94.7%
	Female	16	5.3%
Age (years)	18–40	187	61.7%
	Over 40	115	38.3%
Marital status	Single	95	31.5%
	Married	140	46.3%
	Divorced	60	19.9%
	Widow	7	2.3%
Employment	Unemployed	75	24.8%
	Employed	227	75.2%
	Self-employed	183	60.6%
	Employee	37	12.2%
	Retired	5	1.7%
	Student	2	0.7%
Education^a^	Elementary education	55	18.2%
	Secondary education	217	71.9%
	Higher education	30	9.9%
Monthly income (Tomans)	Low (< 500,000)	148	49%
	Middle (500,000-2,000,000)	130	43.1%
	High (> 2,000,000)	24	7.9%
Age of onset (years) of substance use	Early (< 15)	139	46%
	Late (> 15)	163	54%
History of hospitalization	No	185	61.3%
	Yes; previous psychiatric hospitalization	117	38.7%
Substance use in the family	No	69	22.8%
	Yes	233	77.2%
	One member	98	32.4%
	Two members	59	19.5%
	Three members	34	11.3%
	Four members	18	6%
	Five members	12	4%
	Six members	5	1.7%
	Seven members	7	2.3%
Type of substance use in the family members	Stimulant substances^b^	47	15.6%
	Non-stimulant substances	255	84.4%
Type of substance use in the last year^c^	Tobacco group	290	96%
	Heroin group	216	71.5%
	Opium group	150	49.7%
	Cannabis group	119	39.4%
	Alcohol group	97	32.1%
	Cocaine group	16	5.3%
	Ritalin group	3	1%

^a^Elementary education including illiterate and elementary education; Secondary education including high school education; and Higher education including diploma and higher education

^b^Stimulant substances including Ritalin, amphetamines and cocaine

^c^The tobacco group, including hookah and cigarettes; the heroin group, including heroin and crack; the opium group, including opium and opium sap; the cannabis group, including marijuana and cannabis; the alcohol group, only including alcohol; the cocaine group, only including cocaine; and the Ritalin group, only including Ritalin (methylphenidate)

Based on the demographic characteristics, participants were divided into several groups, including the education level (including three groups, elementary education including illiterate and elementary education, secondary education/high school, and higher educational level including diploma and higher education); the monthly income (including three groups, low-income, middle-income, and high-income); the age of onset of substance use (including two groups, early and late onset); the substance use in the family (including two groups, those with family members who used substances and did not use substances); type of substances used in the family members (including two groups, the family members who used stimulant substances including Ritalin, amphetamines and cocaine, and the family members who used non-stimulant substances); the substance type in the last year (the tobacco group including hookah and cigarette, heroin group including heroin and crack, opium group including opium and sap, cannabis group including marijuana and cannabis, alcohol group only including alcohol, cocaine group only including cocaine, and Ritalin group only including Ritalin (methylphenidate)).

**Fig. 1:**
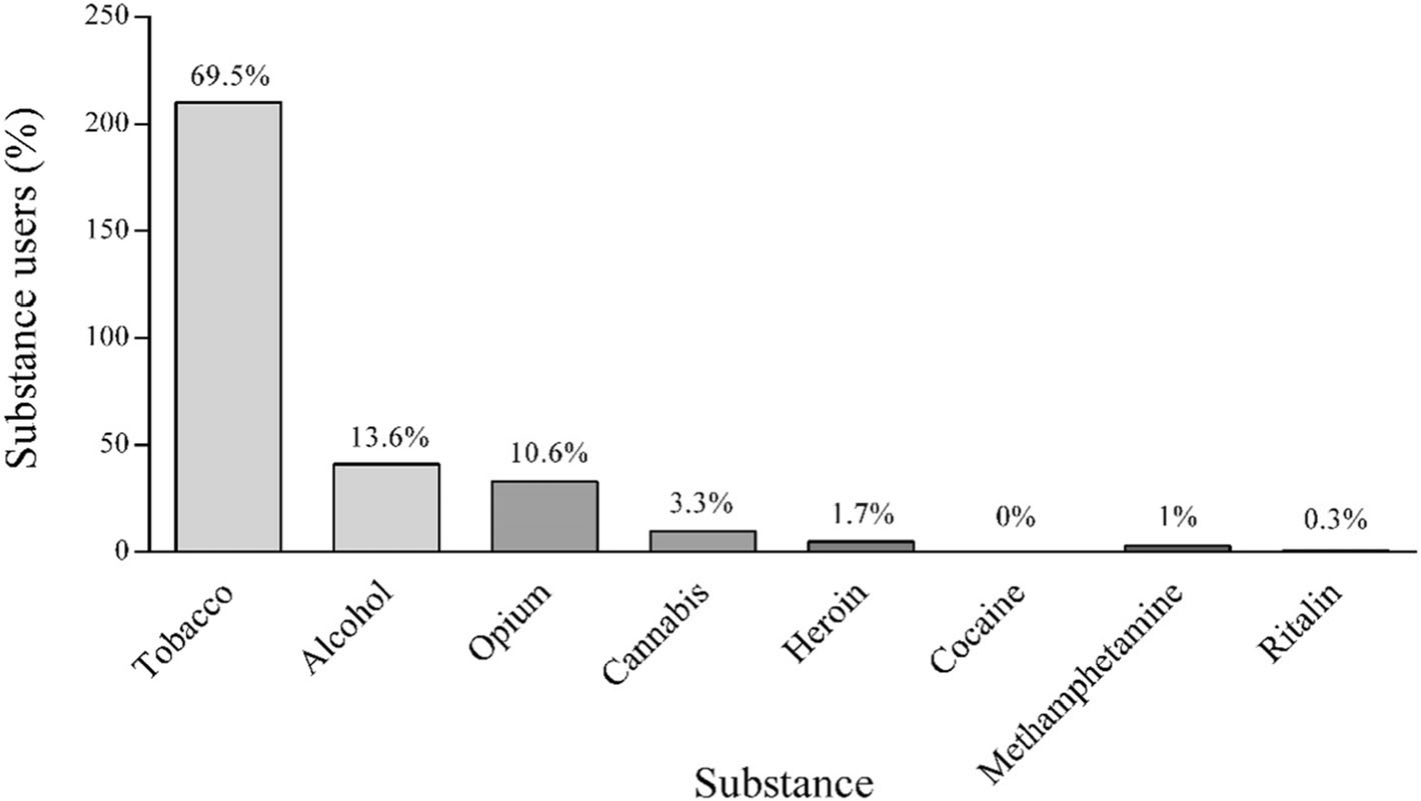
Frequency of the first substance used among the patients who used methamphetamine (MA)

As shown in Fig. 1, tobacco, as the first substance or starting substance, exhibited the most widely-used substance (69.5% of the cases), showing statistically significant differences (*p* ≤ 0.0001). After tobacco, 13.6 and 10.6% of the patients who used MA started substance use with alcohol and opium, respectively, showing statistically significant differences (*p* ≤ 0.0001). Other substances used included cannabis (3.3%), heroin (1.7%), and MA (1%), respectively. Only one case (0.3%) mentioned Ritalin as the first substance and no one (0%) started substance use with cocaine; in total, 93.71% of the patients who used MA started substance use with tobacco, alcohol, or opium.

The comparison between heroin and MA use showed that 41 of the patients who used MA started with MA earlier than heroin (13.58% of all the patients who used MA and 17.7% of the patients who used both heroin and MA). In contrast, 190 of the patients who used MA started with heroin earlier than MA (62.91% of all the patients who used MA and 82% of the patients who used both heroin and MA).

The comparison between cannabis and opium use demonstrated that 92 of the patients who used MA started with cannabis earlier than opium (30.46% of all the patients who used MA and 56.72% of the patients who used both cannabis and opium). In contrast, 70 of the patients who used MA started with opium earlier than cannabis (23.18% of all the patients who used MA and 43.2% of the patients who used both cannabis and opium).

The comparison between cannabis and tobacco use revealed that 167 of the patients who used MA started with tobacco earlier than cannabis (55.30% of all the patients who used MA and 93.29% of the patients who used both cannabis and tobacco). In contrast, 12 of the patients who used MA started with cannabis earlier than tobacco (3.97% of all the patients who used MA and 6.07% of the patients who used both cannabis and tobacco).

The comparison between alcohol and tobacco use indicated that 154 of the patients who used MA started with tobacco earlier than alcohol (50.99% of all the patients who used MA and 80.2% of the patients who used both alcohol and tobacco). In contrast, 38 of the patients who used MA started with alcohol earlier than tobacco (12.58% of all the patients who used MA and 19.79% of the patients who used both alcohol and tobacco).

The comparison between alcohol and opium use showed that 136 of the patients who used MA started with alcohol earlier than opium (45.03% of all the patients who used MA and 73.91% of the patients who used both alcohol and opium). In contrast, 48 of the patients who used MA started with opium earlier than alcohol (15.89% of all the patients who used MA and 26.08% of the patients who used both alcohol and opium).

The comparison between alcohol and cannabis use showed that 100 of the patients who used MA started with alcohol earlier than cannabis (33.11% of all the patients who used MA and 72.99% of the patients who used both alcohol and cannabis). In contrast, 37 of the patients who used MA started with cannabis earlier than alcohol (12.25% of all the patients who used MA and 27% of patients who used both alcohol and cannabis).

A variety of sequencing patterns was obtained in this study, the most common of which included:

Sequencing D: tobacco-opium-heroin-MA (*n* = 24).

Sequencing F: tobacco-alcohol-cannabis-opium-heroin-MA (*n* = 20).

Sequencing C: tobacco-opium-MA (*n* = 19).

Sequencing E: tobacco-alcohol-opium-heroin-MA (*n* = 17).

Sequencing A: MA (*n* = 3).

Sequencing B: tobacco-MA (*n* = 2).

Based on the age of onset of substance use, as mentioned above, patients were divided into two groups, including early onset and late onset; the comparison between sequencing patterns and the age of onset of substance use showed that sequencing F was significantly lower than sequencings C, D, and E (Table 2).

**Table 2:** Relationship between different sequencing patterns and age of onset of substance use

Sequence	C (***n*** = 19)^**a**^	D (***n*** = 24)^**a**^	E (***N*** = 17)^**a**^	F (***N*** = 20)^**b**^	*P-value
Age started (mean ± SD)	16.15 ± 4.34	17.04 ± 4.92	17 ± 3.77	12.05 ± 3.94	< 0.0001

C (sequencing C): tobacco-opium-MA; D (sequencing D), tobacco-opium-heroin-MA; E (sequencing E): tobacco-alcohol-opium-heroin-MA; and F (sequencing F), tobacco-alcohol-cannabis-opium-heroin-MA.

*MA* methamphetamine

Dissimilar values (superscripts a and b) of each row are significantly different.

*Values were analyzed by one-way analysis of variance (ANOVA); values are mean ± SD

The comparison between the age of onset of substance use and current monthly income showed the number of patients who used MA with early onset of substance use was significantly higher in the low-income group (*P*-value = 0.006) (Table 3).

**Table 3:** Relationship between the age of onset of substance use and monthly income

Monthly income	Low-income	Middle-income	High-income	Chi-square	Df	*P-value
**Early onset**	76	44	19	10.146	2	0.006
**Late onset**	67	81	15			

* The chi-square test was used for the statistical differences

The comparison between the age of onset of substance use and education level showed that the number of patients who used MA with high education was lower in the early-onset group, while the number of patients who used MA with primary education was higher in the early-onset group. These findings were statistically significant (*P*-value = 0.04).

The comparison between the age of onset of substance use and the starting substance type showed no significant relationship. Table 4 indicates the mean age of patients who used MA in starting groups with different substances. The highest and lowest mean age was associated with MA (30 years) and tobacco (15.25 years) starting groups, respectively.

**Table 4:** The mean age of patients who used MA in starting groups

First Substance	Mean ± SD
**Opium**	18.06 ± 7.41
**Methamphetamine**	30 ± 14.93
**Cannabis**	16.54 ± 4.03
**Heroin**	19.83 ± 9.78
**Alcohol**	15.79 ± 4.92
**Tobacco**	15.25 ± 4.26

The comparison between substance use in the family and different sequences of substance use showed a higher rate of odds ratio (OR) for sequencing C, as a result of which early onset of substance use was correlated with family member uses. In other words, patients who used MA with family history of substance use started MA use earlier than others.

The comparison between different sequences of substance use and the number of family members who used substances showed that only sequencing F were statistically significant (*P* value = 0.03) (Table 5). The family history of substance use was found to be a risk factor for sequencing F (OR = 0.8).

**Table 5:** Relationship between drug sequences and family history of substance use

Sequencing patterns	Family members with substance use	Family members with no substance use	chi-square	*P-value	OR
N (%)	N (%)	N (%)			
C	13 (68.42)	6 (31.58)	0.92	0.24	1.14
D	17 (70.83)	7 (29.17)	0.75	0.26	1.109
E	14 (82.35)	3 (17.65)	0.22	0.45	0.94
F	19 (95)	1 (5)	3.81	0.03	0.8

C (sequencing C): tobacco-opium-MA; D (sequencing D), tobacco-opium-heroin-MA; E (sequencing E): tobacco-alcohol-opium-heroin-MA; and F (sequencing F), tobacco-alcohol-cannabis-opium-heroin-MA.

*MA* methamphetamine

*OR* odds ratio

* The chi-square test was used for the statistical differences

The relationship between the use of stimulants in the family, including cocaine, MA, and Ritalin, showed no significant difference between sequences and use of stimulants in the family. No significant relationship was found between employment status and the age of onset of substance use as well as between different substance use and marital status.

## Discussion

A number of studies have investigated the sequence of substance use initiation [20–23], suggesting that initiation of a substance and the sequence patterns of the substance use may be a risk factor for other behaviors. Fewer studies have investigated the sequence of substance use initiation for MA. The present study evaluated the sequential patterns of substance use before MA use disorders in Iran.

Our findings showed early onset of MA use in Iran. This early-onset MA use was significantly higher in those with lower income, primary education, no history of hospitalization, and family history of substance use. Tobacco followed by alcohol and opium, as starting substances, was found to be an important risk factor for MA use disorders. Sequencing D (tobacco-opium-heroin-MA) was the most common sequencing pattern in patients who used MA. Sequencing F (tobacco-alcohol-cannabis-opium-heroin-MA) was significantly related to the early onset of MA use.

The abuse of substances, including MA, is associated with substantial costs and limited treatment options. Researches on the patterns and sequences of substance initiation provide a far more comprehensive portrait to develop suitable prevention and early intervention strategies.

In the present study, tobacco, followed by alcohol and opium, was found to be the first substance or starting substance used in patients with MA use, serving as a starting substance for initiation to MA use. Tobacco, alcohol and opium accounted for 93.71% of the first substances used by patients who used MA participated in this study. In a study carried out by *Yen* et al. in Taiwan, cigarette was found to be the most commonly used substance, and most adolescents had used cigarette, alcohol, areca quid, and glue before MA use [24]. No differences were found between cigarette smoking and alcohol use in their study with the present study; however, glue and areca quid are not used in Iran. In a study performed by *Brecht* et al. in California, tobacco, alcohol, and marijuana were found as a sequencing pattern for MA use in 95% cases [9]. The difference between marijuana and opium use seems to be due to the prevalence of opium use in Iran. Only 3 patients who used MA started with MA use (0.99%) in this study, which was consistent with the study conducted by *Brecht* et al. in California [9].

Prevalence of substance use in the last year was as follows:

1. The tobacco group, including hookah and cigarettes: 96% of the patients who used MA
2. The heroin group, including heroin and crack: 71.6% of the patients who used MA
3. The opium group, including opium and opium sap: 49.7% of the patients who used MA
4. The cannabis group, including marijuana and cannabis: 39.3% of the patients who used MA
5. The alcohol group, only including alcohol: 32.1% of the patients who used MA
6. The cocaine group, only including cocaine: 5.3% of the patients who used MA
7. The Ritalin group, only including Ritalin (methylphenidate): 1% of the patients who used MA

The prevalence of substance use in the past year was similar to that found in the study carried out by *Darke* et al.in 2012 [10].

In this study, 46.1% of the people who used MA started before 15 years, while 53.9% of them started MA use after 15 years; the mean age of onset of the first substance use was 15.9 years. In the study carried out in Taiwan, 71% of the patients who used MA used substances after 15 years of age [24]; the reason for this difference can be attributed to the cohort effect, because the study of *Yen* et al. in Taiwan was carried out in 2005 while our study was performed in 2016, and the age of onset of substance use decreases over time. In a study conducted by *Darke* et al.in Australia [10], the mean age of the onset of substance use was 14 years, which was consistent with our findings.

Commonly-used sequencing patterns obtained in this study included D (tobacco-opium-heroin-MA; *n* = 24), F (tobacco-alcohol-cannabis-opium-heroin-MA; *n* = 20), C (tobacco-opium-MA; *n* = 19), E (tobacco-alcohol-opium-heroin-MA; *n* = 17), A (MA; *n* = 3), and B (tobacco-MA; n = 2), respectively. Common sequencing patterns obtained in the study carried out by *Yen* et al. in Taiwan [24] included MA as an initiator (*n* = 12), cigarette-MA (*n* = 86), cigarette-areca quid-MA (*n* = 41), cigarette-alcohol-MA (*n* = 39), alcohol-cigarette-MA (*n =* 12), cigarette-areca quid-alcohol-MA (*n =* 39), cigarette-alcohol-areca quid-MA (*n =* 39), and alcohol-cigarette-areca quid-MA (*n* = 9). The difference between the sequence patterns can be attributed to the high prevalence of opioid use in Iran and the high prevalence of other substances such as Areca quid in Taiwan. However, the first 4 sequencing patterns showing high prevalence started with cigarettes both in the present study and in the study conducted in Taiwan [24].

Most patients who used MA participated in this study used heroin before MA (82%), but in the study carried out in Taiwan [24], none of the patients who used MA used heroin before MA, which could be due to the difference in the age of patients who used MA. In the study carried out in Taiwan [24], patients who used MA participated in that study were adolescents with 19 years or younger. In addition, previous studies showed that heroin is generally used in older adults [25]. Importantly, there are studies showing that the tendency to use different substances changes at different times and in different societies.

The comparison between the sequencing patterns of tobacco and cannabis use indicated that most patients who used MA (93.29%) used tobacco earlier than cannabis, which was consistent with the study conducted by *Darke* et al. in Australia [10]. Inconsistent with these findings, the study conducted with *Brecht* et al. in California [9] showed that tobacco, marijuana, and alcohol acted as a sequential substance to MA use, in which alcohol was the most frequent substance to be used. This can be attributed to the frequency of alcohol availability and use as well as the increasing prevalence of marijuana use in recent years [26].

The comparison between the sequencing patterns and the age of onset of substance use showed that sequencing F (tobacco-alcohol-cannabis-opium-heroin-MA) was significantly related to early onset of substance use. This means that patients who used MA with lower age of onset used more substances before starting MA use. This finding was consistent with the studies carried out in Taiwan [24] and Australia [10], demonstrating that early onset of substance use has been associated with poly-drug abuse in the future.

In this study, there was a significant relationship between early onset of substance use and education; early onset of substance use was found in the patients who used MA with low educational levels while late onset of substance use was found in those with higher educational levels. In a study performed by *Russell* et al., a significant relationship was found between MA use and lower education, showing that patients who used MA generally had a lower education level [5]. The highest and lowest mean ages of onset of substance use were found to be 30 and 15.25 years in patients who used MA and tobacco, respectively; the mean ages of onset of opium, cannabis, alcohol, and heroin use were 18.68, 16.44, 15.59, and 19.83 years old, respectively. The mean ages of onset found in this study were lower as compared with the study conducted in California [9], in which patients who used alcohol had the lowest mean age, and patients who used heroin, followed by MA, had the highest mean age. The reason for the higher mean age of patients who used alcohol in our country and the present study can be attributed to religious beliefs about alcohol use.

In the present study, early onset of MA use was associated with a family history of substance use; in other words, patients who used MA with a family history of MA use started MA use earlier than other substances. In addition, sequencing F was also significantly associated with the greater number of family members with a history of substance use. In the studies carried out by *Russell* et al. [5] and *Svingen* et al. in Nebraska [27], a significant relationship was found between the family history of substance use and substance use in patients who used substances. The relationship between a family history of substance use and the age of onset of substance use showed that patients who used MA with a family history of substance use started to use substances 3.8 years earlier [28]; the results from these two studies were consistent with the results from this study.

The early age of onset of substance use was significantly associated with low monthly income. In most similar studies, the role of income levels was not investigated because those studies have chosen their samples from adolescents and only one study was presented descriptive income sources. In the study carried out by *Darke* et al. in Australia [10], 88, 21, and 7% of the patients who used MA derived their primary income from the government, criminal activity, and wages, respectively. In collection, the early age of substance use leads to less education, in turn resulting in lower-paid jobs.

To the best of our knowledge, this is the first study to examine the sequencing patterns of MA use, which can be the basis for future studies. However, the present study had some potential limitations, including the use of self-reporting, social desirability, selection bias and the low number of participants, especially female gender; the latter limitation led to no gender comparisons for variables. Therefore, the results may limit the generalizability of the results to some extent.

## Conclusions

The present study is the first study to examine the sequential patterns of substance use for MA disorders in Iran. In this study, the early-onset MA use was significantly higher in patients who had lower income, primary education, no history of hospitalization, and family history of substance use. Tobacco, alcohol and opium were found to be as a sequential pattern for initiation to MA use.

Tobacco-alcohol-cannabis-opium-heroin-MA sequencing was significantly related to the early onset of the MA use.

Findings from this study may support targeted prevention efforts and development of more effective interventions. Standardized measures to decrease and control access to main starting and sequencing substances, including tobacco, alcohol, and opium, can not only greatly help decrease the early onset of the MA use, but also lead to development of suitable prevention and early intervention strategies. In addition, programs focusing on increasing knowledge and changing the attitudes of adolescents toward drug abuse can assist to prevent MA use through decreased use of these substances in the society. However, because of gender limitations, it is recommended to include more women users in future studies in order to make gender comparisons possible.

## Data Availability

The datasets used and/or analyzed during the current study are available from the Shiraz University of Medical Sciences on reasonable request.

## Abbreviation

MA: Methamphetamine
